# Factors on Quality of Life Improvement in Septorhinoplasty: Prospective Evaluation Using the Functional Rhinoplasty Outcome Inventory 17 and Its Minimally Important Difference

**DOI:** 10.1007/s00266-026-05796-1

**Published:** 2026-04-21

**Authors:** Georg-Philipp Kuehl, Olcay Cem Bulut, Patrick J. Schuler, Lukas Fiedler, Frank Riedel, Ingo Baumann, Ralph Hohenberger

**Affiliations:** 1 Department of Otorhinolaryngology Head and Neck Surgery, Heidelberg University Hospital, Im Neuenheimer Feld 400, 69120 Heidelberg, Germany; 2 HNO-Zentrum Rhein-Neckar, Stresemannstraße 22, 68165 Mannheim, Germany; 3 Department of Otorhinolaryngology, Head and Neck Surgery, SLK-Kliniken Am Gesundbrunnen 20-26, 74078 Heilbronn, Germany

**Keywords:** Quality of life, Septorhinoplasty, Functional Rhinoplasty Outcome Inventory, BDDQ-AS, Body dysmorphic disorder, FROI-17, MCID

## Abstract

**Background:**

Septorhinoplasty (SRPL) is a frequently performed surgery aimed to improve health-related quality of life (HRQOL), typically assessed using disease-specific questionnaires. This study aimed to analyze the factors influencing postoperative HRQOL improvement and to establish the minimal clinically important difference (MCID) for the Functional Rhinoplasty Outcome Inventory (FROI-17).

**Methods:**

In this prospective study, pre- and twelve months postoperative, disease-specific HRQOL was assessed in 95 patients undergoing functional SRPL (Level of Evidence III). Clinical parameters including age, sex, surgical approach, type of nasal deformity, revision surgery and screening on body dysmorphic disorder were obtained. The primary endpoint was the change in FROI-17 scores. MCID was calculated with an internal anchor.

**Results:**

Mean FROI-17 scores improved from 38.0 ± 16.0 to 24.5 ± 20.0 (*p* < 0.001). Uni- and multivariate analysis identified the preoperative FROI-17 as the strongest predictor of QoL improvement (*p* < 0.001). Only the preoperative nasal shape (*p* = 0.02) and the BDD screening result (*p* = 0.04) also showed significant correlations in the univariate analysis. The subcategory “self-consciousness" of the FROI-17 showed high sensitivity and specificity for the BDD screening results. An MCID of 13.4 points was calculated, matching the mean postoperative improvement in this cohort.

**Conclusions:**

The preoperative FROI-17 score serves as a reliable predictor of postoperative HRQOL improvement following SRPL. These findings underscore the value of implementing patient-reported outcome measures such as the FROI-17 in preoperative counseling to enhance transparency and long-term patient satisfaction.

**Level of Evidence III:**

This journal requires that authors assign a level of evidence to each article. For a full description of these Evidence-Based Medicine ratings, please refer to the Table of Contents or the online Instructions to Authors www.springer.com/00266.

This study included 95 patients undergoing functional septorhinoplasty, their health-related quality of life was measured with the Functional Rhinoplasty Outcome Inventory (FROI-17) and improved from 38.0 ± 16.0 to 24.5 ± 20.0 (*p* < 0.001) one year after surgery

- Uni- and multivariate analysis identified the preoperative FROI-17 as the strongest predictor of QoL improvement (*p* < 0.001). Preoperative nasal shape (*p* = 0.02) and the body dysmorphic disorder screening result (*p* = 0.04) also showed significant correlations in the univariate analysis
- The subcategory “self-consciousness" of the FROI-17 showed good sensitivity and specificity for the screening results of body dysmorphic disorder
- The minimal clinically important difference (MCID) was calculated to be 13.4, matching the mean improvement in the study cohort

## Background

Deformities of the inner and outer nose can limit health-related quality of life (HRQOL) due to functional problems such as impaired nasal breathing and aesthetic dissatisfaction with the nasal appearance. Functional septorhinoplasty (SRPL) addresses both aspects and is a common surgery performed by ENT, maxillofacial and plastic surgeons. HRQOL evaluation and improvement have been established as key endpoints in patients undergoing SRPL [1, 2]. Several disease-specific patient reported outcome measures (PROMs) have been developed and validated, the most suitable being the rhinoplasty outcomes evaluation (ROE), standardized cosmesis and health nasal outcomes survey (SCHNOS) and the Functional Rhinoplasty Outcome Inventory (FROI-17), which takes functional aspects more into account [3]. Postoperative QOL improvements in disease-specific and general HRQOL have been demonstrated in recent years [4], yet some patients remain unsatisfied after surgery. Single factors and their unbeneficial effect on postoperative QOL gain have been identified, like symptoms of body dysmorphic disorder (BDD) or the nasal shape [5–7]. It would therefore be of great value to patients and surgeons to identify individuals at a higher risk of postoperative dissatisfaction when considering surgical intervention. This assessment may involve clinical characteristics as well as PROM results. To enhance the interpretation of studies involving PROMs, the concept of the minimal clinically important difference (MCID) has been introduced. It refers to the smallest change in a PROM score that is perceived by patients as beneficial (or harmful) [8]. Though its use in various studies, no MCID has been established for the FROI-17. The aim of this study was therefore to prospectively assess HRQOL in a large cohort of patients undergoing functional SRPL, and to identify relevant predictors of HRQOL improvement using the FROI-17 in both univariate and multivariate analyses. In addition, the MCID was calculated to support the interpretation of FROI-17 scores and to quantify the effect of surgical interventions in this and future studies.

## Patients and Methods

### Study Design and Aim

This prospective study was conducted at the Heidelberg University Hospital to analyze factors on the patients’ quality of life gain. Patients were invited preoperatively to participate voluntarily in the study. In case of participation, clinical data, a screening questionnaire on body dysmorphic disorder and the FROI-17 were obtained. Patient data were pseudonymized and anonymized for the analysis. Participation in the study had no impact on treatment, and withdrawal was possible at any time without consequences. Twelve months postoperatively, FROI-17 scores were evaluated again in a clinical follow-up.

### Patients and Data Collection

Between May 2017 and June 2022, 95 patients were included in the study. All patients undergoing functional SRPL, regardless of other clinical characteristics, were asked to voluntarily participate in the study and, in case of informed consent, were included in the study. The preliminary calculation yielded approximately 100 patients to obtain significant results. Exclusion criteria were incomplete FROI-17 questionnaires, insufficient knowledge of German or an age under 18 years at the time of surgery. SRPL was scheduled if an objective nasal deformity was present (deviated nose, humped nose or a combination of both or other deformities, e.g., saddle nose) and the patients desired both functional and aesthetic improvements. The type of deformity was categorized by the respective surgeon. Data collection included age, sex, surgical approach, previous nasal surgeries and nasal shape. The primary endpoint was the difference between pre- and post-operative mean scores of the FROI-17.

### Questionnaires

FROI-17: This questionnaire consists of 17 questions; each can be answered on a Likert-scale from 0 to 5 points. A higher score indicates a greater impairment of HRQOL. The total score is scaled from zero to 100 points [9]. Further subcategory can be calculated: nasal symptoms (items 1–7), general symptoms [8–14], and self-confidence [15, 16]. E.g., “self-confidence” consists of two questions (“low self-esteem" and “I am embarrassed by my nasal shape”) with a range of zero to ten points. The 17th question is a global question about the shape and function of the nose.

BDDQ-AS: The BDDQ-AS is a screening tool for symptoms of BDD in septorhinoplasty patients [10]. It consists of seven symptoms and has a binary screening result as positive or negative. It has been validated in multiple languages including German [11]. A positive result is not equivalent to the diagnosis of this condition. In case of positive screening, dysmorphic concerns were adequately addressed and further discussed; psychological treatment was provided when in doubt. Surgery was only scheduled, when an objective deformity of the nose was present, contradicting the actual diagnosis of BDD, defined as a psychiatric condition with high occupation with slight or imagined defects in one’s physical appearance causing relevant distress in the daily life [12].

### Statistics

The data analysis was performed using IBM SPSS Statistics 29.0.2.0. A *p* value ≤ 0.05 was considered significant. The univariate analysis was performed with Student’s *t* test, the multivariate analysis with backward multivariate linear regression models.

MCID calculation, anchor-based method: As external anchor, all patients were asked to rate the subjective result one year after surgery on a five-point Likert-scale (“As a result of the surgery I feel…”: much worse; worse; the same; much better). As an internal anchor, the last question of the FROI-17 was used (“overall impairment by the nose, shape and function”). Because of its holistic approach, including both shape and function of the nose and the prospective data collection without the risk of a recall bias, it was considered the optimal anchor for the MCID calculation. The respective differences in pre- and postoperative scores of this item in each patient results in an 11-point Likert scale. A correlation of these differences in the anchor with the changes in the FROI total score (as dependent variable) of at least 0.3 is recommended [13, 14]. The MCID was then defined as the mean difference in the FROI-17 total score between zero points difference and an improvement by one point in the internal anchor.

### MCID Calculation, Distribution-Based Method

For better interpretation of the anchor-based MCID, a calculation of the MCID with the distribution-based method and a standard deviation of 0.5 was included.

Dealing with missing values: The BDDQ-AS screening result was missing in 13 of 95 patients. All datasets of the other parameters were complete. The missing values were tested for Missing Completely at Random (Little test: *p* = 0.124). Missing values were excluded in the multivariate analysis by complete-case analysis, resulting in *n* = 82. For the univariate analysis, pairwise exclusion was used where possible to obtain more data, resulting in *n* = 82 for the BDDQ-AS analysis and *n* = 95 for all other variables.

## Results

Ninety-five patients were included in this study; the patient's characteristics and clinical details of the performed surgeries are summarized in Table 1. Most patients (87.4%) underwent primary SRPL, and most patients presented with a (hump-)deviated nose (70.5%). The non-deviated nose deformities included hump nose (*n* = 11), saddle nose (*n* = 5), tip deformities (*n* = 5), deformities after orthognathic surgery (*n* = 4), nasal valve collapse (*n* = 2) and broad nose (*n* = 1). Patients reported a significant improvement of their HRQOL by 13.5 ± 21.4 points measured with the FROI-17 when comparing pre- and twelve months postoperative mean scores (FROI-17 total score: 38.0 ± 16.0 [95% CI 35–41.5] to 24.5 ± 20.0 [CI 20.5–28.6], *p* < 0.001).

**Table 1:** Clinical characteristics of the study cohort

	Absolute frequency	Relative frequency (%)
Patients	95	100
Sex		
Female	58	61.1
Male	37	38.9
Age		
Mean	30.4	
Median	27	
Minimum	18	
Maximum	69	
Surgical approach		
Closed	43	45.3
Open	52	54.7
fSRPL revision		
No	83	87.4
Yes	12	12.6
Nasal deformity		
Non-deviated nose Hump nose Other shape disorders	28 11 17	29.5 11.6 17.9
Deviated nose Deviated nose Hump-deviated nose	67 30 37	70.5 31.6 38.9
BDDQ-AS screening		
Valid	82	86.3
Negative	52	63.4
Positive	30	36.6

*SRPL* functional septorhinoplasty, *BDDQ-AS* body dysmorphic disorder concern questionnaire—aesthetic surgery

The baseline HRQOL did only differ slightly in the subgroups gender (female: 38.9 ± 16.4 vs. male: 36.5 ± 15.4, *p* = 0.59) and approach (open: 38.8 ± 16.1 vs. closed: 37 ± 16, *p* = 0.60). In patients undergoing primary surgery, the baseline HRQOL was lower (38.9 ± 15.7), compared to revision SRPL (32.0 ± 16.8, *p* = 0.15), and also patients with deviated nose (40.4 ± 15.7 vs. non-deviated: 32.2 ± 15.3 *p* = 0.02) and positive BDD screening (42.4 ± 17.1 vs. negative screening: 35.1 ± 14.9, *p* = 0.05) showed lower HRQOL scores.

### Univariate Analyses

**Fig. 1:**
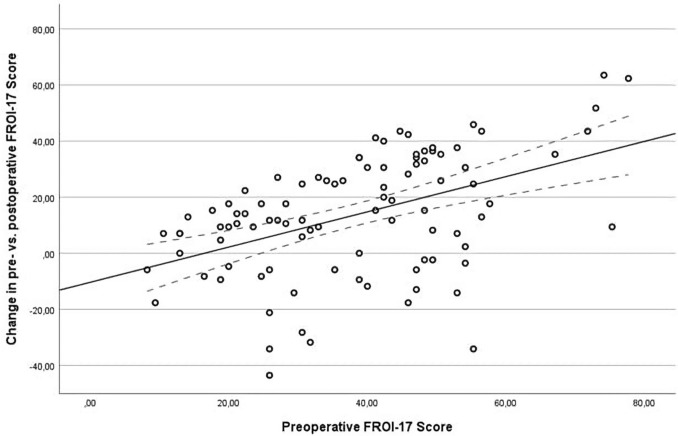
Scatter plot of preoperative FROI-17 total scores and mean postoperative improvement. *FROI* Functional Rhinoplasty Outcome Inventory 17. The dotted line shows the 95% confidence interval of the regression line (formula y= -9.97 + 0.62 * *x*)

Univariate analyses showed that in all categories and respective specifications the FROI score improved in the postoperative measurement (Table 2). Patients undergoing revision surgery, as well as those with non-deviated nasal shapes, showed smaller improvements compared to their counterparts, though these differences did not reach statistical significance. Significant predictors of HRQOL improvement included nasal shape (deviated vs. non-deviated), BDDQ-AS screening result, and preoperative FROI-17 score. The preoperative FROI-17 total score showed linear relationship (*r* = 0.46, *p* < 0.001) with the improvement in the FROI-17 score; the higher the preoperative score, the greater the difference as depicted in Fig. 1.

**Table 2:** Univariate analysis of surgery-related factors, their respective changes in the FROI-17 total scores and significance of the factors in simple linear regression

Factor		*N*	Preoperative (±SD)	Postoperative (±SD)	Change (±SD)	Significance of change	Significance of factor	Cohen’s *d* [95% CI]
Sex	Female	58	38.9 (16.4)	24.4 (20.0)	14.5 (21.0)	*p* < 0.001	*p* = 0.55	0.13 [− 0.19, 0.44]
	Male	37	36.5 (15.4)	24.8 (20.2)	11.8 (22.3)	*p* < 0.001		
Age		95					*p* = 0.24	
Surgical procedure	Closed	43	37.0 (16.0)	23.0 (17.9)	14.1 (21.5)	*p* < 0.001	*p* = 0.80	0.06 [− 0.32, 0.43]
	Open	52	38.8 (16.1)	25.8 (21.6)	12.9 (21.6)	*p* < 0.001		
SRPL revision	No	83	38.9 (15.7)	24.4 (20.6)	14.4 (21.8)	*p* < 0.001	*p* = 0.26	0.34 [− 0.30, 0.98]
	Yes	11	32.0 (16.8)	25.0 (15.9)	7.0 (22.0)	*p* = 0.10		
Deviated nose	No	28	32.2 (15.3)	26.3 (21.1)	5.9 (21.1)	*p* = 0.07	***p*** = **0.02**	0.51 [0.10, 0.91]
	Yes	67	40.4 (15.7)	23.8 (19.6)	16.6 (20.9)	*p* < 0.001		
BDDQ-AS	Negative	52	35.1 (14.9)	25.0 (17.9)	10.1 (18.2)	*p* < 0.001	***p*** = **0.04**	0.48 [0.02, 0.94]
	Positive	30	42.4 (17.1)	22.4 (20.8)	20.0 (24.8)	*p* < 0.001		
Preoperative FROI total score		95					***p*** < **0.001**	

Bold values indicate statistical significance (*p* < 0.05)

*FROI* Functional Rhinoplasty Outcome Inventory 17, *SRPL* functional septorhinoplasty, *BDDQ-AS* body dysmorphic disorder concern questionnaire – aesthetic surgery

The subcategory self-confidence of the FROI-17 consists of two questions that can be graded from 0 to five points each. It showed a strong association with the BDD screening results. With a combined score of 7 or higher in this subcategory, 14 of 15 patients had a positive screening in the BDDQ-AS (sensitivity = 93%). With a score of two or lower, 26 of 27 patients were negative in the BDDQ-AS (specificity = 96%). Consistently, the mean scores in the subcategory self-confidence differed significantly depending on the BDDQ-AS results (positive: 37.0 ± 40.3, negative: 7.1 ± 25.1, *p* < 0.001).

### Multivariate Analyses

Multivariate analyses with all factors included showed the preoperative FROI-17 total score as the only significant and most relevant predictor on HRQOL improvement (Table 3). Nasal shape and BDD screening were retained as potentially relevant variables, but they did not reach statistical significance, likely due to collinearity with the baseline FROI-17 score.

**Table 3:** Multivariate regression model including all factors in HC4-method

Model	Factor	Regression coefficient	Sig.	Sig. of the model	*corr. R*^2^ * 100	*f*^*2*^
1	Sex	− 0.6	0.89	< **0.001**	24.9%	0.33
	Age	− 0.1	0.64			
	Surgical approach	0.3	0.94			
	SRPL revision	1.3	0.83			
	Deviated nose	8.4	0.13			
	BDDQ-AS	3.6	0.46			
	Preop. FROI-17 score	0.6	**< 0.001**			
2	Sex	− 0.6	0.89	**< 0.001**	25.9%	0.35
	Age	− 0.1	0.65			
	SRPL revision	1.3	0.83			
	Deviated nose	8.3	0.14			
	BDDQ-AS	3.7	0.44			
	Preop. FROI-17 score	0.6	**< 0.001**			
3	Age	− 0.1	0.61	**< 0.001**	26.9%	0.36
	SRPL revision	1.4	0.81			
	Deviated nose	8.3	0.14			
	BDDQ-AS	3.8	0.42			
	Preop. FROI-17 score	0.6	**< 0.001**			
4	Age	− 0.1	0.63	**< 0.001**	27.8%	0.38
	Deviated nose	8.1	0.13			
	BDDQ-AS	3.8	0.42			
	Preop. FROI-17 score	0.6	**< 0.001**			
5	Deviated nose	8.4	0.11	**< 0.001**	28.3%	0.39
	BDDQ-AS	4.0	0.39			
	Preop. FROI-17 score	0.6	**< 0.001**			
6	Deviated nose	9.0	0.08	**< 0.001**	28.4%	0.39
	Preop. FROI-17 score	0.6	**< 0.001**			
7	Preop. FROI-17 score	0.7	**< 0.001**	**< 0.001**	26.8%*	0.36

Bold values indiacte statistical significance (*p* < 0.05)

^*^= Uncorrected *R*^2^ as single factor

*Sig.* significance, *f*^2^ Cohen’s effect size, *corr.* corrected, *FROI* Functional Rhinoplasty Outcome Inventory 17, *SRPL* functional septorhinoplasty, *BDDQ-AS* body dysmorphic disorder concern questionnaire – aesthetic surgery

Therefore, a backward multivariate regression model without the preoperative FROI-17 score as factor was calculated (Table 4). Only the nasal shape remained as a significant predictor. However, in excluding the preoperative FROI-Total score, this second model showed consistently small effect sizes. All other factors showed no significant association to HRQOL improvement in the multivariate analysis.

**Table 4:** Backward multivariate regression models without the preoperative FROI-17 score

Model	Factor	Regression coefficient	Sig.	Sig. of the model	*corr. R*^*2*^ * 100	*f*^*2*^
1	Sex	− 2.3	0.64	0.09	6.3%	0.06
	Age	− 0.1	0.73			
	Surgical approach	1.4	0.77			
	SRPL revision	− 2.0	0.77			
	Deviated nose	13.1	**0.01**			
	BDDQ-AS	6.6	0.20			
2	Sex	− 2.1	0.67	0.05	7.4%	0.08
	Age	− 0.1	0.67			
	Surgical approach	1.4	0.77			
	Deviated nose	13.3	**0.01**			
	BDDQ-AS	6.6	0.19			
3	Sex	− 2.0	0.68	**0.03**	8.5%	0.09
	Age	− 0.1	0.69			
	Deviated nose	13.1	**0.01**			
	BDDQ-AS	6.9	0.16			
4	Sex	− 2.2	0.63	**0.01**	9.5%	0.10
	Deviated nose	13.4	**0.01**			
	BDDQ-AS	6.9	0.16			
5	Deviated nose	13.1	**0.01**	**0.005**	10.4%	0.11
	BDDQ-AS	7.5	0.11			
6	Deviated nose	14.7	**0.004**	**0.004**	9.8%*	0.10

Bold values indiacte statistical significance (*p* < 0.05)

^*^= Uncorrected *R*^2^ as single factor

*Sig.* significance, *f*^2^ Cohen’s effect size, *corr.* corrected, *FROI* Functional Rhinoplasty Outcome Inventory 17, *SRPL* functional septorhinoplasty, *BDDQ-AS* body dysmorphic disorder concern questionnaire – aesthetic surgery screening

In conclusion, the preoperative FROI-17 score proved to be the strongest predictor of postoperative HRQOL gain. Nasal shape and body image concerns were additional relevant factors but are associated with the preoperative FROI-17 scores.

### MCID Calculation

The external anchor inquiring about the subjective improvement after surgery correlated poorly with the mean difference in the pre- vs. postoperative FROI scores (*r* = 0.26, *p* = 0.03) and was therefore withdrawn.

The 17th question of the FROI as internal anchor showed a satisfactory correlation with the mean change in the FROI-17 total scores with *r* = 0.55 (*p* < 0.001). The difference in mean scores between no difference and an improvement by one point on the 11-point Likert scale resulted in a calculated MCID of 13.4 (± 16.8). The mean improvement in the FROI-17 in this cohort (13.5 ± 21.4) matches the calculated MCID. Forty-seven of 89 patients (52.8%) achieved an improvement above this threshold (six patients had initial FROI scores below 13.4 and were unable to reach this value). The standard deviation of the mean FROI improvement was 21.4. Multiplied with 0.5, the MCID using the distribution-based method was 10.7.

## Discussion

### Key Findings

This prospective study evaluated 95 patients and examined clinical factors associated with the postoperative improvement in health-related quality of life (HRQOL) following functional septorhinoplasty (SRPL), using the FROI-17 as a disease-specific PROM. A mean improvement of 13.5 ± 21.4 points was observed, confirming the positive impact of SRPL on HRQOL. The preoperative FROI-17 total score emerged as the strongest predictor of postoperative improvement. Additionally, a deviated nose and a positive result on the body dysmorphic disorder (BDD) screening were significantly associated with HRQOL improvement in univariate analysis but also associated with the preoperative FROI-17 score. Other factors—including age, sex, surgical approach, and revision status—showed no significant impact. The calculated minimal clinically important difference (MCID) of 13.4 points closely matched the observed mean improvement, supporting its validity and underscores the practical relevance for the interpretation of the FROI-17 in functional SRPL.

### Strengths and Weaknesses

A key strength of this study is its prospective design, allowing for systematic data collection and minimizing recall bias. Additionally, the inclusion of a broad range of clinical factors in the analysis of HRQOL provides a comprehensive understanding of potential predictors. The use of a validated, disease-specific PROM (FROI-17), combined with a relatively large patient cohort, enhances the robustness and generalizability of the findings. Notably, to the best of our knowledge, this is the first study to establish a minimal clinically important difference (MCID) for the FROI-17, thereby improving the interpretability of results in both this and future studies. It must be noted though, that the calculated MCID in this cohort, exactly matching the mean improvement, may underlie the effect of overfitting to the underlying dataset, and results may differ in other populations.

However, several limitations should be acknowledged. First, the exclusive use of the FROI-17 as the sole instrument for HRQOL assessment may introduce bias, particularly due to its emphasis on functional rather than aesthetic outcomes. The use of a complimentary PROM focusing rather on the aesthetic dimensions of SRPL like the SCHNOS or ROE would have taken this aspect more into account. As a result, the findings of this study apply mainly to patients with a clear functional indication, particularly those with nasal deviation, who showed the greatest improvement. Second, the number of cases included in the multivariate analysis was reduced due to missing data. For the univariate analysis, pairwise exclusion was used where it was possible to obtain more data. Although this limits the transferability of the results, it reduces the loss of cases and increases the statistical power. Nevertheless, the missing data may have affected the statistical power and reliability of some associations. Third, the postoperative data collection may have been subject to response bias, particularly from patients who were either highly satisfied or dissatisfied. Also, a possible selection bias caused by the exclusion of patients with incomplete questionnaires or language barriers may have skewed the results. Finally, the use of the difference between pre- and postoperative scores as the primary endpoint could lead to different conclusions than analyses based solely on postoperative scores, as baseline symptom severity heavily influences the magnitude of observed change. Also, the postoperative HRQOL may be subject to change, and the stability of improvements beyond the observed 12 months remains uncertain.

### Current Literature

Patients with a deviated nose showed worse preoperative and better postoperative HRQOL compared to non-deviated noses and hence a higher improvement in the FROI-17 scores. These findings are consistent with the existing literature when functional outcomes are used as endpoints [6]. Yet, when examining factors on aesthetic satisfaction with the ROE, a preoperative deviated nose may result in similar or even worse HRQOL [7, 15]. This discrepancy may be explained by the fact that nasal breathing tends to be more severely impaired in deviated noses, which contributes to a greater functional burden. At the same time, achieving satisfactory aesthetic correction of a deviated nose poses a greater surgical challenge, potentially limiting perceived aesthetic success.

The prevalence of 36.6% of BDDQ-AS positive patients in this study matches the results of previous studies [5, 10, 16, 17]. These patients showed higher pre- and lower postoperative mean scores in the FROI-17, resulting in a higher difference and higher HRQOL gain in the FROI-17. Given the fact that an established BDD is a contraindication for any aesthetic surgery [12], these results seem paradox [5]. Previous work has shown that surgery can have a positive effect on HRQOL even when mild forms or only symptoms of BDD are revealed, especially if the symptoms are limited to a clearly defined feature like the shape of the nose [16, 18–20]. Recent findings by Declau et al. suggest that the BDDQ-AS may not specifically measure clinical BDD, but rather general dissatisfaction or psychosocial distress related to nasal appearance [21]. The significant postoperative reduction in positive screening rates indicates that the tool may reflect concerns that are responsive to surgery. This underlines the importance of interpreting BDDQ-AS results cautiously and not as a definitive contraindication for rhinoplasty. The postoperative results in patients screened positive for BDD may also be influenced by the indication for the surgery, in this cohort mainly functional, and contrary results in populations undergoing aesthetic SRPL and the use of a PROM with an aesthetic focus. Both implications of a positive BDD screening, possibly higher HRQOL gain due to worse preoperative perception and on the other hand possibly exclusion from surgery due to manifest BDD, have to be carefully considered in the individual case.

It is essential to recognize that screening positive on the BDDQ-AS is not equivalent to receiving a clinical diagnosis of BDD. The relatively high prevalence of positive screening results likely reflects a strong preoccupation with nasal appearance rather than meeting the full diagnostic criteria for BDD. According to standard definitions, BDD is characterized by excessive concern over minor or imagined defects that cause significant distress or impairment. In this study, all patients presented with clear functional nasal deformities, which would typically exclude a diagnosis of BDD. Moreover, the FROI-17 emphasis on functional outcomes, and aesthetic concerns influence the total score only partially. Thus, improvements in FROI-17 scores among patients with BDD symptoms are understandable, especially when functional outcomes improve despite residual aesthetic concerns [20]. Still, patients with psychiatric symptoms such as BDD tend to report lower HRQOL related to nasal function and appearance overall [5, 16, 22].

Given the high prevalence of BDD symptoms and associated psychiatric comorbidities, surgeons should remain vigilant during preoperative evaluation. PROMs and validated screening tools for BDD, such as the BDDQ-AS, are valuable and should be incorporated into routine assessment [20]. This is further supported by the strong sensitivity and specificity of the FROI-17 subdomain “self-consciousness” with proposed thresholds of ≥7 and ≤2 points serving as indicators for positive and negative BDDQ-AS screenings, respectively. This subdomain is easy to apply and interpret in clinical settings. In cases of doubt, dysmorphic concerns should be explored in detail with the patient, and referral to psychological evaluation should be considered before proceeding with surgery.

In this study, neither sex nor age had a significant effect on postoperative HRQOL improvement. This is consistent with several previous studies showing that functional and aesthetic improvements after SRPL are largely independent of these factors [23–25]. Additionally, no significant differences in HRQOL improvement were observed between open and closed surgical approaches, reflecting current literature with equivalent outcomes between the two techniques [26, 27].

Both primary and revision SRPL led to improvements in FROI-17 scores. However, revision status was not a significant predictor of HRQOL gain in this cohort, possibly due to the small number of revision cases (*n* = 11), limiting the statistical power. Other studies have confirmed that revision procedures can improve HRQOL; however, patients undergoing revision are more likely to require additional surgeries and may be less satisfied overall. Lower satisfaction rates—particularly regarding aesthetic outcomes—have been reported in revision patients compared to those undergoing primary SRPL [25, 28].

## Conclusion

This study confirms the beneficial effect of functional SRPL on the HRQOL. The preoperative FROI-17 score emerged as the strongest predictor for postoperative improvement. The presence of a deviated nose and a positive BDD screening were further factors impacting the HRQOL in univariate analyses but were associated with the preoperative FROI-17 scores. The FROI-17 subcategory “self-consciousness” shows high sensitivity and specificity for the BDD screening, adding to the benefits of its routine use in preoperative patient evaluation. In any case, the results of BDD screening must be interpreted in conjunction with all patient characteristics to avoid misinterpretation. The MCID was determined to be 13.4 points, closely aligning with the observed mean improvement in this cohort.

## References

[1] Chen K, Zhou L. The effect of functional rhinoplasty on quality of life: A systematic review and meta-analysis. Aesthetic Plast Surg. 2024;48(5):847–54. 37173413 10.1007/s00266-023-03390-3

[2] Bulut OC, Wallner F, Oladokun D, Kayser C, Plath M, Schulz E, et al. Long-term quality of life changes after primary septorhinoplasty. Qual Life Res. 2018;27(4):987–91. 29204784 10.1007/s11136-017-1761-8

[3] Xiao H, Zhao Y, Liu L, Xiao M, Qiu W, Liu Y. Functional/aesthetic measures of patient satisfaction after rhinoplasty: A review. Aesthet Surg J. 2019;39(10):1057–62. 30722008 10.1093/asj/sjz029

[4] Bulut OC, Wallner F, Plinkert PK, Prochnow S, Kuhnt C, Baumann I. Quality of life after septorhinoplasty measured with the functional rhinoplasty outcome inventory 17 (FROI-17). Rhinology. 2015;53(1):54–8. 25756079 10.4193/Rhino14.008

[5] Hohenberger R, Endres P, Salzmann I, Plinkert PK, Wallner F, Baumann I, et al. Quality of Life and screening on body dysmorphic disorder, depression, anxiety in septorhinoplasty. Laryngoscope. 2024;134(5):2187–93. 38050954 10.1002/lary.31212

[6] Bulut OC, Wallner F, Hohenberger R, Plinkert PK, Baumann I. Quality of life after primary septorhinoplasty in deviated- and non-deviated nose measured with ROE, FROI-17 and SF-36. Rhinology. 2017;55(1):75–80. 28025985 10.4193/Rhin16.243

[7] de Souza OE, Lavinsky-Wolff M, Migliavacca RO, de Azeredo AM, Colognese Gabbardo AV, Velho JS. Analysis of determinants of postoperative satisfaction after rhinoplasty. Laryngoscope. 2022;132(8):1569–75. 34716715 10.1002/lary.29923

[8] Jaeschke R, Singer J, Guyatt GH. Measurement of health status. Ascertaining the minimal clinically important difference. Control Clin Trials. 1989;10(4):407–15. 2691207 10.1016/0197-2456(89)90005-6

[9] Bulut C, Wallner F, Plinkert PK, Baumann I. Development and validation of the functional rhinoplasty outcome inventory 17 (FROI-17). Rhinology. 2014;52(4):315–9. 25479208 10.4193/Rhino13.098

[10] Lekakis G, Picavet VA, Gabriëls L, Grietens J, Hellings PW. Body dysmorphic disorder in aesthetic rhinoplasty: Validating a new screening tool. Laryngoscope. 2016;126(8):1739–45. 27223322 10.1002/lary.25963

[11] Hohenberger R, Baumann I, Krisam R, Wallner F, Plinkert PK, Lippert BM, et al. Validating the body dysmorphic disorder questionnaire-aesthetic surgery in a German rhinoplasty population. J Plast Reconstr Aesthet Surg. 2022;75(2):893–939. 34670729 10.1016/j.bjps.2021.09.029

[12] Phillips KA. Body dysmorphic disorder: common, severe and in need of treatment research. Psychother Psychosom. 2014;83(6):325–9. 25322928 10.1159/000366035

[13] Revicki D, Hays RD, Cella D, Sloan J. Recommended methods for determining responsiveness and minimally important differences for patient-reported outcomes. J Clin Epidemiol. 2008;61(2):102–9. 18177782 10.1016/j.jclinepi.2007.03.012

[14] Klukowska AM, Vandertop WP, Schröder ML, Staartjes VE. Calculation of the minimum clinically important difference (MCID) using different methodologies: case study and practical guide. Eur Spine J. 2024;33(9):3388–400. 38940854 10.1007/s00586-024-08369-5

[15] Cingi C, Eskiizmir G. Deviated nose attenuates the degree of patient satisfaction and quality of life in rhinoplasty: a prospective controlled study. Clin Otolaryngol. 2013;38(2):136–41. 23406156 10.1111/coa.12099

[16] de Souza TSC, Patrial M, Meneguetti AFC, de Souza MSC, Meneguetti ME, Rossato VF. Body dysmorphic disorder in rhinoplasty candidates: prevalence and functional correlations. Aesthetic Plast Surg. 2021;45(2):641–8. 32875438 10.1007/s00266-020-01930-9

[17] AlAwadh I, Bogari A, Azhar T, AlTaylouni N, AlSughier N, AlKarzae M, et al. Prevalence of body dysmorphic disorder among rhinoplasty candidates: a systematic review. Ear Nose Throat J. 2021;103(6):377–83. 34789021 10.1177/01455613211056543

[18] Felix GAA, de Brito MJA, Nahas FX, Tavares H, Cordás TA, Dini GM, et al. Patients with mild to moderate Body Dysmorphic Disorder may benefit from rhinoplasty. J Plast Reconstr Aesthet Surg. 2014;67(5):646–54. 24508222 10.1016/j.bjps.2014.01.002

[19] Rabaioli L, Oppermann PO, Pilati NP, Klein CFG, Bernardi BL, Migliavacca R, et al. Evaluation of postoperative satisfaction with rhinoseptoplasty in patients with symptoms of Body Dysmorphic Disorder. Braz J Otorhinolaryngol. 2022;88(4):539–45. 32978118 10.1016/j.bjorl.2020.07.013 PMC9422649

[20] Crerand CE, Phillips KA. Reply to: “Patients with mild to moderate body dysmorphic disorder may benefit from rhinoplasty.” J Plast Reconstr Aesthet Surg. 2014;67(12):1754–5. 24997505 10.1016/j.bjps.2014.06.013

[21] Declau F, Pingnet L, Smolders Y, Fransen E, Verkest V. The body dysmorphic disorder questionnaire-aesthetic surgery: Are we screening the troublesome patients? Facial Plast Surg. 2024;40(5):571–80. 38198825 10.1055/a-2241-9934

[22] Hohenberger R, Baumann I, Riedel F, Plinkert PK, Bulut OC. Impact of psychiatric symptoms on nasal perception in septorhinoplasty patients. Facial Plast Surg. 2024;40(5):560–4. 38196074 10.1055/a-2240-8943

[23] AlHarethy S, Al-Angari SS, Syouri F, Islam T, Jang YJ. Assessment of satisfaction based on age and gender in functional and aesthetic rhinoplasty. Eur Arch Otorhinolaryngol. 2017;274(7):2809–12. 28417237 10.1007/s00405-017-4566-z

[24] Nuyen B, Spataro EA, Olds C, Kandathil CK, Most SP. Relationship of sociodemographic factors and outcomes in functional rhinoplasty. Facial Plast Surg. 2019;35(1):85–9. 30654390 10.1055/s-0039-1677708

[25] Riedel F, Wähmann M, Bran GM, Conder M, Bulut OC. Quality of life after functional aesthetic septorhinoplasty in primary surgery vs. revision surgery-a monocentric study. HNO. 2019;67(3):192–8. 30132128 10.1007/s00106-018-0554-x

[26] Gupta R, John J, Ranganathan N, Stepanian R, Gupta M, Hart J, et al. Outcomes of closed versus open rhinoplasty: a systematic review. Arch Plast Surg. 2022;49(5):569–79. 36159386 10.1055/s-0042-1756315 PMC9507448

[27] Metin M, Avcu M. The effect on patient satisfaction of the postoperative nasal topographic, demographic, and functional results of open and closed septorhinoplasty techniques. J Craniofac Surg. 2021;32(3):868–73. 33038179 10.1097/SCS.0000000000007120

[28] Spataro E, Piccirillo JF, Kallogjeri D, Branham GH, Desai SC. Revision rates and risk factors of 175–842 patients undergoing septorhinoplasty. JAMA Fac Plast Surg. 2016;18(3):212–9. 10.1001/jamafacial.2015.2194 PMC5600890 26967651

